# Experimental Verification of Thermal Insulation in Timber Framed Walls

**DOI:** 10.3390/ma15062040

**Published:** 2022-03-10

**Authors:** Daniela Michálková, Pavol Ďurica

**Affiliations:** Department of Building Engineering and Urban Planning, University of Zilina, 010 26 Žilina, Slovakia; pavol.durica@uniza.sk

**Keywords:** timber framed, wall, relative humidity, thermal conductivity, material properties

## Abstract

Current environmental crisis calls for sustainable solutions in the building industry. One of the possible solutions is to incorporate timber-framed constructions into designs. Among other benefits, these structures are well established in many countries, originating in traditional building systems. This paper focuses on experimental timber-frame walls. Different wall assemblies vary in thermal insulation materials and their combinations. We investigated ten experimental wall structures that have been exposed to natural external boundary conditions since 2015. The emphasis was on their state in terms of visual deterioration, mass moisture content, and thermal conductivity coefficient. We detected several issues, including defects caused by inappropriate realization, causing local moisture increase. Material settlement in loose-fill thermal insulation was another issue. Concerning was a significant change in the thermal conductivity of wood fiber insulation, where the current value almost doubled in one case compared to the design value determined by the producer.

## 1. Introduction

The current environmental crisis is triggering scientists worldwide to develop more sustainable solutions in the building industry. At first glance, the easiest way is to return to traditional and thus natural materials and incorporate them into the design [1,2,3].

To provide more sustainable housing options while respecting increasingly challenging legislative requirements [4], timber-frame constructions are gaining popularity [5,6]. They are lightweight structures, suitable for most climate conditions. Their advantage in terms of high thermal resistance while maintaining relatively small thickness is undeniable. With these structures, we can minimize wall thickness while maintaining the material in perfect condition in case of future recycled use. Moreover, wood is, by proper management, a renewable natural source that requires minimum primary energy [7].

Our university department has focused on these structures for more than ten years, studying their behavior in terms of heat and mass flows. A standard user is mainly unable to determine the state of built-in materials and is often unaware of several defects causing more or less severe complications. Several studies focus on non-destructive methods to determine structure conditions [8,9,10]. However, they can be misleading. We took the liberty of our research and took the experimental walls apart to analyze the state of loadbearing studs and thermal insulation.

Our experimental walls serve to study possibilities in thermal insulation combinations and order and their influence on temperature and moisture flux within the wall. Our wall fragments fall into the category of timber-frame constructions, with load-bearing timber studs and various types of thermal insulation. Half of them are diffusely closed using intelligent climate membrane both from exterior and interior.

Each insulation material is known and well established in the current building design. Mineral fiber—both glass and basalt fiber—is often used for its thermal resistance properties and as a fire retardant and acoustic insulation [11,12,13,14].

Phenolic foam has outstanding thermal properties, known for its multifunction as loadbearing, heat-insulating, and thermal protective material [15].

The representatives for more sustainable solutions are wood fiber and sheep wool insulation. Wood fiber boards are made from softwood, in this case, as boards. They are often used in timber housing, not only as thermal but also acoustic insulation. Wood as a natural hygroscopic material [16] can regulate surrounding air humidity and thus improve the interior environment [17].

Sheep wool is the most traditional of all investigated materials, used as primary or recycled wool after a process of impregnation. According to recent studies, this insulation can compete with other commonly used insulation materials and even outstand them [18,19].

The main goal of this study is to provide objective information about the condition of timber frame walls that can be expected after exposure without any severe maintenance.

## 2. Materials and Methods

Within the University of Žilina, the research team from the Department of Building Engineering and Urban Planning managed to build a pavilion laboratory with the support of projects from the structural funds. The laboratory has been active since 2011. Its detailed description can also be found in the literature [20].

The wall structures evaluated in this research were built in 2015, after a significant laboratory reconstruction and its adaptation to the current market.

### 2.1. Experimental Laboratory

The research currently consists of three rooms, thermally insulated from the rest of the building. One of these rooms focuses on window structures. The other two contain exterior timber-frame walls designed for passive housing. The research in these two rooms focuses on investigating the synergic transfer of heat and water through envelope structures.

**Figure 1:**
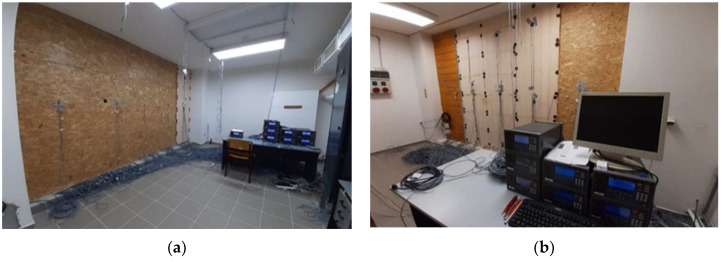
Research laboratory: (**a**) Wall oriented towards east; (**b**) Wall oriented towards south.

**Figure 2:**
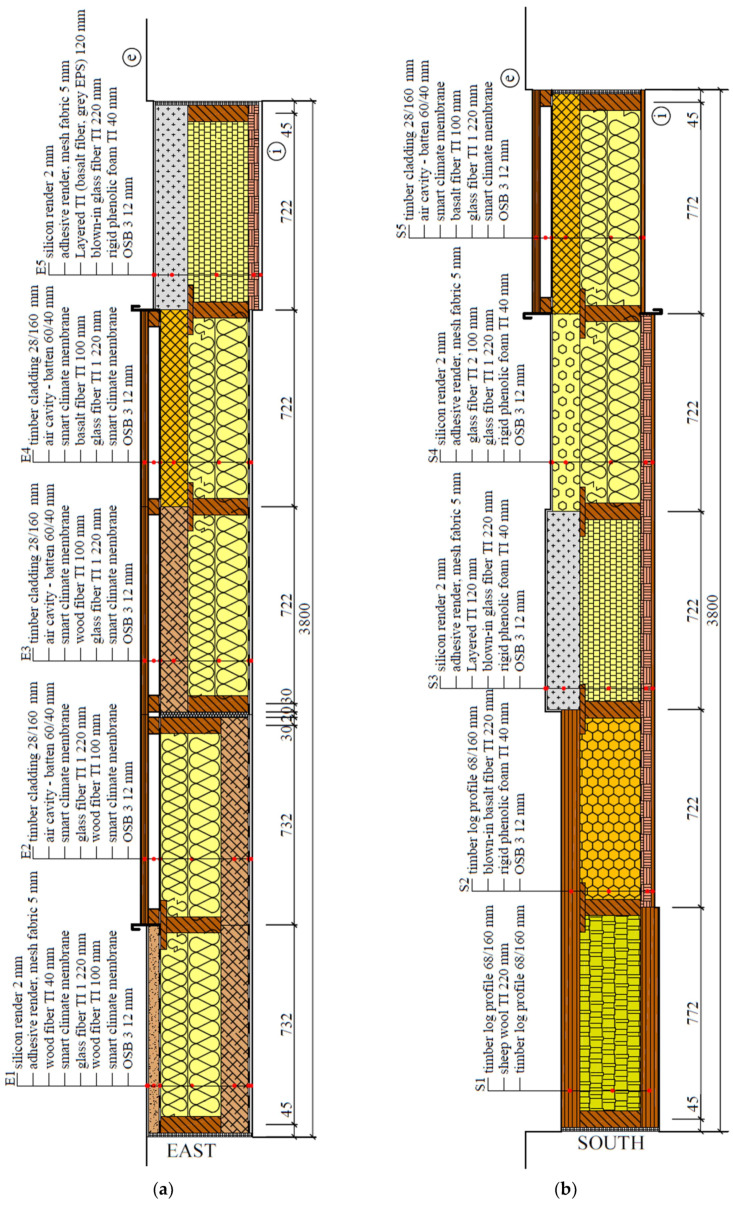
Timber-framed walls of the University of Zilina research center: (**a**) Wall oriented towards east; (**b**) Wall oriented towards south.

The direction of the experimental walls differs. One faces the southeast (15° deviation from the east), the other the southwest (15° deviation from the south) (Figure 1). Each monitored wall consists of five different structures (Figure 2). All ten are timber-framed, using various materials for thermal insulation and its order in-wall depth. Materials and their main physical properties are shown in Table 1. The two fragments are the same in both walls to compare different orientations. This enables us to determine the effect of wall orientation on their behavior.

The experimental samples are within the exterior laboratory wall, enabling exposure to the natural external boundary conditions while ensuring the indoor environment via an HVAC system.

**Figure 3:**
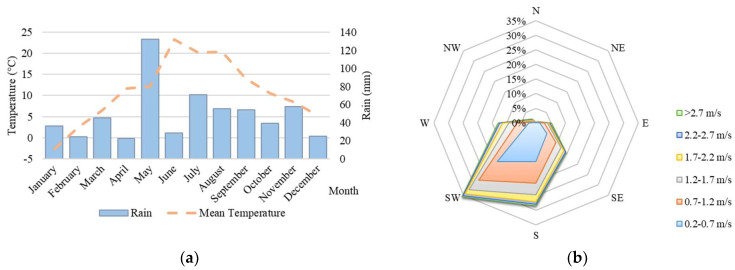
Exterior boundary conditions: (**a**) Rain and mean temperature; (**b**) Wind speed and direction.

Each wall fragment is separately removable for any future research or practice requirements. Temperature and humidity sensors are in three height levels on each structure. Other sensors monitor ambient temperatures and relative humidity. Air conditioning units adjust the indoor environment to a constant value of 20 °C temperature and 50 % relative humidity. The weather station on the laboratory building roof measures the parameters of the outdoor environment. Figure 3 depicts reference boundary conditions for the exterior. The prevalent rain is present in May, while the maximal mean temperature in this region is expected in June. Based on Figure 3b, the predominant wind speed is 2.2–2.7 m/s in the southwest direction.

### 2.2. Verification Methodology

**Figure 4:**
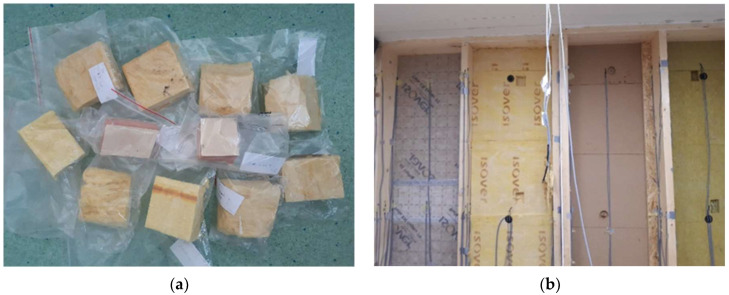
Process of wall disassembly (**a**) Part of samples collected from southern wall; (**b**) Photo of the eastern wall during the disassembly.

During the verification, we carried out three measurement sets. One of them was the mass moisture of the wooden elements—timber studs, wood fiber thermal insulation—performed in situ. The second set focused on thermal insulation from glass fibers and phenolic foam, measuring the mass moisture of collected samples (Figure 4a). The measurements of the thermal conductivity coefficient of thermal insulation comprise the last measurement set. Figure 4b reflects the process of disassembly of the eastern wall.

**Figure 5:**
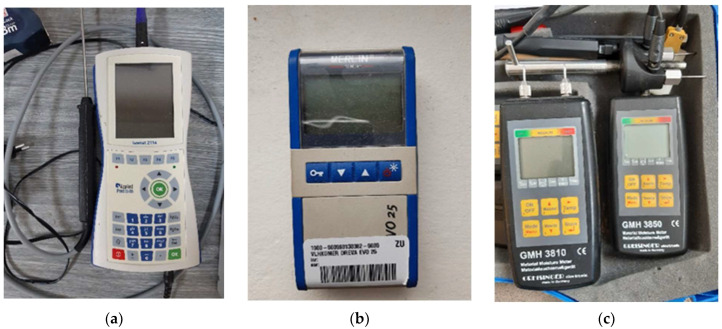
Measuring devices: (**a**) Isomet 2114; (**b**) Merlin EVO25; (**c**) Greisinger GMH 3810 and Greisinger GMH 3850.

The primary device for thermal conductivity measurements was Isomet 2114, with a needle probe displayed in Figure 5a. It operates with a dynamic temperature field based on the pulse method [21].

To ensure the accuracy of the outcomes, we measured the humidity of studs, log profiles, and wood fiber insulation with four devices. Two were capacitive probes for measuring wood moisture Merlin EVO25 (Figure 5b) and Testo. The other two were resistance humidity meters from the company Greisinger in two models—GMH 3810 with grooving tips and GMH 3850 with push-in tips (Figure 5c).

**Figure 6:**
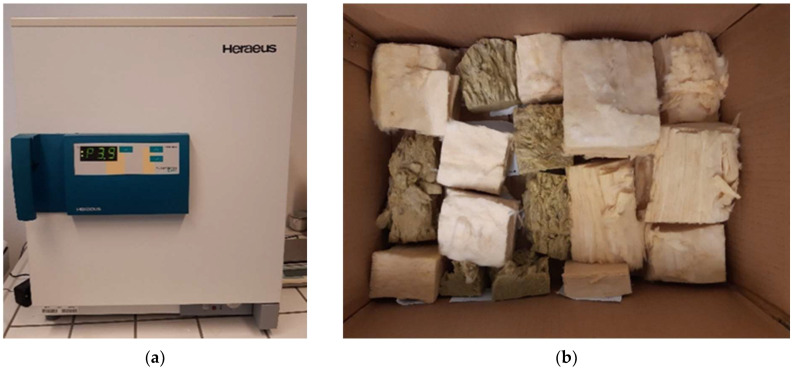
(**a**) Dryer Heraeus Function Line UT6P; (**b**) Samples after reaching a stable weight.

To measure the mass humidity of other materials, we collected samples at three height levels—below the ceiling, in the middle, and approximately 30 cm above the floor. Afterward, we transferred them to another laboratory room equipped with a dryer, ensuring their airtightness. We calculated the mass moisture by the gravimetric method thanks to weighing with an accuracy of 0.01 g and subsequent drying in a Heraeus Function Line UT6P device (Figure 6a) after reaching a stable weight (Figure 6b).

## 3. Results

We divided the results into three subsections. The first describes defects obtained after visual observation. The second depicts the discrepancy between the original and measured values of the thermal conductivity coefficient of thermal insulations. In the last section, we present values of mass moisture both in thermal insulation and timber studs.

### 3.1. Visual Defects

During the inspection, we detected several issues, although most of the walls were in perfect condition. We want to emphasize that all materials used in these walls were new, ready to implement to each structure without any previous damage.

**Figure 7:**
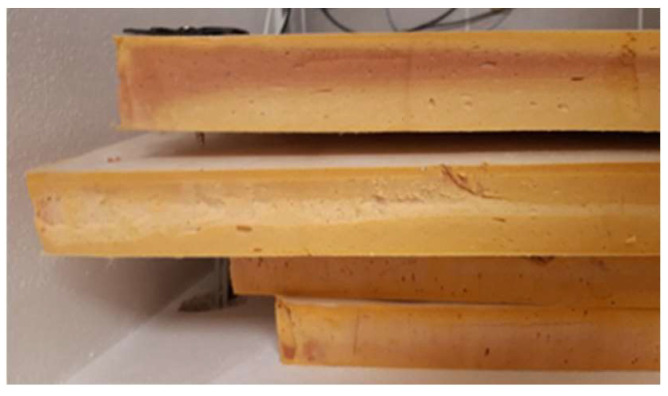
Phenolic foam insulation after color change.

The individual materials did not show any visual changes, with the only exception being Phenolic foam boards. Their original color is pinkish brown. Throughout the years this changed to yellow. This modification is visible in Figure 7, where only the first board partly maintained the original color. However, the mass moisture content was low. Therefore, we concluded that the cause is natural degradation via oxidation.

**Figure 8:**
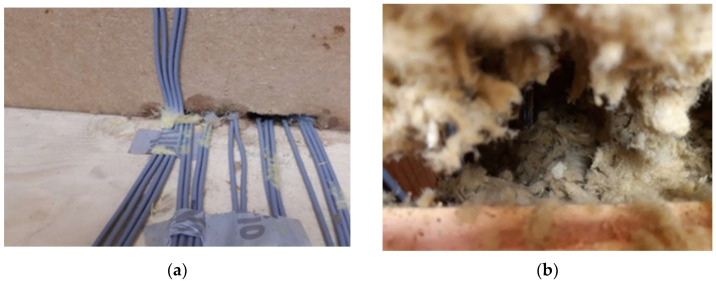
Detected defects: (**a**) Increased moisture transfer in fragment E3; (**b**) Air cavity in the middle of S2.

A more severe defect was detected at the threshold level in fragment E3 in wood fiber insulation. We discovered increased moisture originating in the cable network from the exterior that created a passage for water (Figure 8a). It is a challenging but crucial detail in properly executing weather barriers. In this case the leakage was substantial, while the moisture content measured with Greisinger GMH 3850 reached 18.6%. This problem turned out to only be local, as the boards’ humidity dropped to 10.1%, just 10 cm aside. However, this was the only fragment with such an issue, whereas all of them were built simultaneously by the same team. Therefore, it is not plausible that this would be the only wall with poor execution. More likely, this fact indicated the unsuitability of using this wood fiber material when more crossings are necessary, without the possibility of securing impermeable contact.

We revealed a severe shortcoming in fragments insulated with blown-in thermal insulation (based on mineral fibers—glass and basalt). We detected air cavities that would significantly reduce building energy performance due to the created thermal bridges. The cavities occurred on top of the walls under the ceiling, caused by the settlement. We measured 3 cm in S3, 8 cm in E5 (both glass fiber insulation), and 7 cm in S2 (basalt fiber insulation). However, these are experimental walls with an emphasis on their professional execution. Nevertheless, the cavities occurred not only on the top, but also in the middle of the structures’ height. It occurred in the place of necessary wiring. Although the wire was thin, it prevented the blown material from filling the lower part sufficiently, thus creating a cavity visible in Figure 8b.

### 3.2. Coefficient of Thermal Conductivity

**Figure 9:**
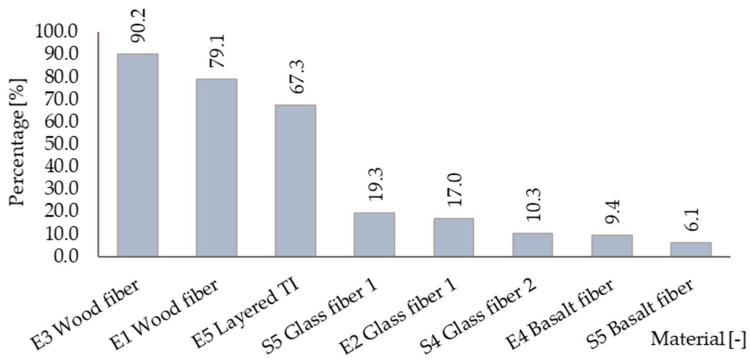
Percentage of thermal conductivity coefficient elevation in thermal insulation materials.

Table 2 presents the thermal conductivity coefficient, measured directly in-situ with an Isomet 2114 with a needle probe and their original design values. It also shows the percentage difference between the two values. Figure 9 displays this difference in a graphical form.

The most significant discrepancy was allocated in wood fiber insulation. The value almost doubled in fragment E3 due to the hygroscopic nature of the material. As stated in the previous section, we discovered imperfections in the weather barrier layer, which unavoidably leads to higher moisture content, causing a change in the thermal conductivity coefficient. We also detected a more than 65% increase in layered thermal insulation, consisting of 30 mm basalt fiber insulation combined with 90 mm of grey polystyrene. The other materials based on mineral fibers showed lower alterations than those mentioned above, thermal insulation of basal fibers being superior from this point of view.

We want to emphasize that our goal is not to compare these materials with each other. These materials have different properties and are built in diverse wall fragments combined with various materials, thus having different exposure conditions. Therefore, this should be received only as a presentation of measured data individually for each sample.

### 3.3. Mass Moisture

**Figure 10:**
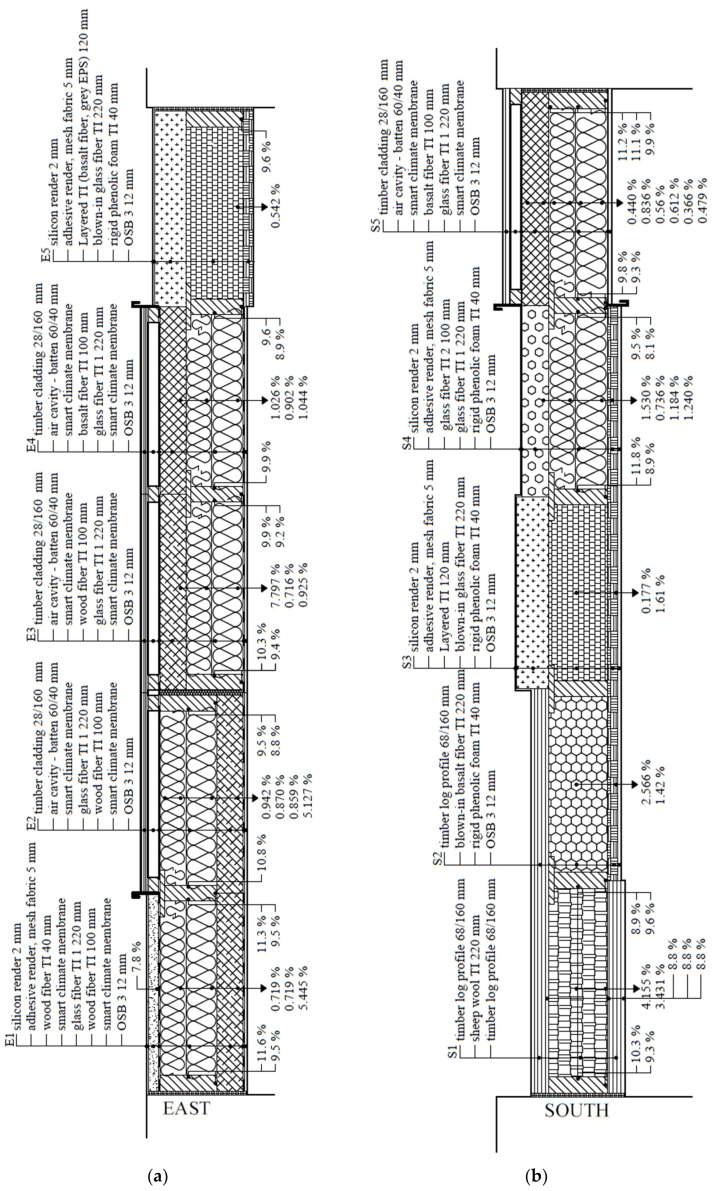
Mass moisture of individual materials: (**a**) Wall oriented towards east; **(b**) Wall oriented towards south.

As mentioned, mass moisture of massive wooden components was measured in-situ with laboratory equipment. For other materials, the moisture was stated through simple calculations using their weight before and after the drying process. Figure 10 shows all obtained data. Values from calculations are marked with an arrow.

Sample collection and the in-situ measurements took place in three different height levels. Figure 10 represents only the maximum values of each material.

**Figure 11:**
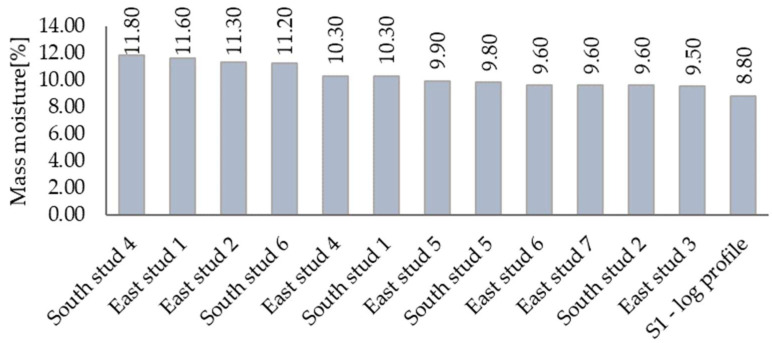
Mass moisture of built-in timber profiles.

Figure 11 shows all measured data concerning mass moisture of timber components. Noteworthy is that the maximal value was 11.8%, indicating no potential danger in terms of mold growth or load-bearing capacity.

**Figure 12:**
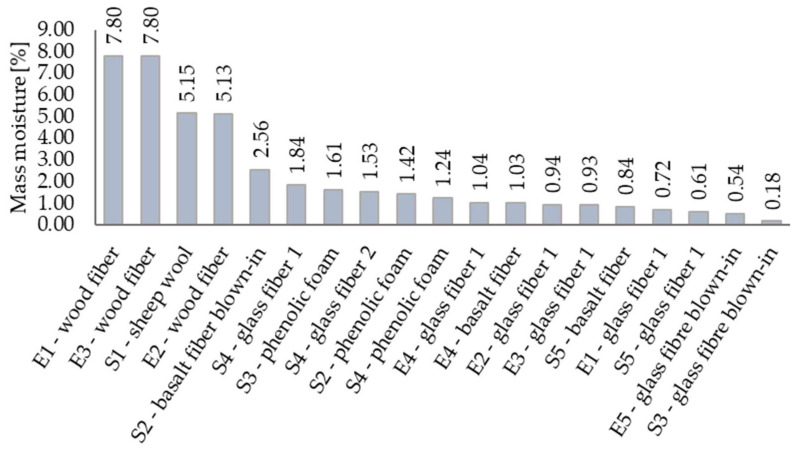
Mass moisture of built-in thermal insulation.

Separately, Figure 12 depicts the mass moisture of each thermal insulation. As indicated from previous sections, wood fiber insulation in fragments reached higher values of mass moisture, even though these are both diffusely closed wall assemblies.

## 4. Discussion

During our research, we obtained a wide variety of data based on measurements of thermal conductivity coefficient and mass moisture. We could monitor several issues, often caused by the material placement.

The essential part of any building—the load-bearing structure—was faultless in terms of visual defects or increased moisture content. The maximum mass moisture was 11.8%, which means that there are no conditions supporting mold creation and development or any other moisture-related issue [22].

According to expectations, mineral fiber insulation remained in perfect condition. It is an inorganic and thus very durable material. The only issue occurred in fragments with loose filling. Even though many producers recommend this blown-in insulation for exterior walls, we would not support this statement. In our case, the execution was professional, focused only on three fragments. Nonetheless, the contractors could not ensure long-term, lasting conditions without additional thermal bridges caused by material settlement, often occurring by this material [23,24]. As a result, this could cause high thermal losses and eventually material degradation due to mold formation on the interior surface due to low surface temperature.

The hygroscopic nature of wood fiber insulation, emphasized in previous studies [16,17], turned out to be a disadvantage. The results showed a significant dependency between mass moisture and thermal conductivity coefficient, causing discrepancies between design and actual values. According to our measurements, the value of the thermal conductivity coefficient throughout the years almost doubled—from value 0.045 to value 0.86—increasing by 90%. This factor further influences the thermal resistance of the whole building envelope, thermal losses, and thus final energy consumption. Another important discovery was the results in fragment E3 with penetrated climate membrane. The influence that this slight imperfection had on the whole layer was so significant that we would not advise using this material from the exterior side of the wall.

The fragment S1 with sheep wool insulation proved to be a reliable assembly, even though it is the only wall consisting solely of natural materials—log profiles from both sides, sheep wool in-between. Neither timber studs nor timber log profiles showed higher moisture content. The sheep wool itself was in excellent condition and could compete with newly established wall structures.

This research provided a broad investigation of timber-framed walls after long-term exposure to natural exterior conditions. Compared to non-destructive methods often used for evaluation, we contributed with more reliable outcomes. It is in our best interest to continue with pending research and gradually provide additional information.

## Figures and Tables

**Table 1:** Materials and their main physical properties.

Material	ρ ^1^ [kg/m^3^]	λ ^2^ [W/(m^2^∙K)]	μ ^3^ [–]	c ^4^ [J/(kg∙K)]
Wooden cladding	400	0.180	157	2510
Silicon render	1600	0.860	130	920
Adhesive render with mesh fabric	1660	0.900	20	900
Timber log profile	400	0.180	157	2510
Smart climate membrane	364	0.350	100,000	1470
Wood fiber thermal insulation (TI)	265	0.480	5	2100
Glass fiber TI 1	64	0.030	1	940
Glass fiber TI 2	148	0.034	1	1030
Basalt fiber TI	100	0.036	1	1020
Blown-in glass fiber TI	35	0.043	1	940
Blown-in basalt fiber TI	50	0.040	1	1020
Layered TI—30 mm basalt fiber and 90 mm grey polystyrene	25	0.033	30	1100
TI—rigid phenolic foam	35	0.021	35	1400
TI—sheep wool	16	0.042	1.5	1720
OSB 3	650	0.130	50	1700

^1^ ρ—bulk density; ^2^ λ—thermal conductivity coefficient; ^3^ µ—water vapor diffusion resistance factor; ^4^ c—specific heat capacity.

**Table 2:** The shift in thermal conductivity coefficient.

Material	Wall	λ before [W/(m^3^∙K)]	λ after [W/(m^2^∙K)]	Change Percentage [%]
Wood fiber T I ^1^	E1	0.045	0.081	79.1
	E3		0.086	90.2
Glass fiber TI ^1^ 1	E2	0.030	0.035	17.0
	S5		0.036	19.3
Glass fiber TI ^1^ 2	S4	0.034	0.038	10.3
Basalt fiber TI ^1^	E4	0.036	0.039	9.4
	S5		0.038	6.1
Layered TI ^1^	E5	0.033	0.055	67.3

^1^ TI stands for thermal insulation.

## Data Availability

Not applicable.

## References

[1] Suhamad D.H., Martana S. (2020). Sustainable Building Materials. IOP Conf. Ser. Mater. Sci. Eng..

[2] Švajlenka J., Kozlovská M. (2018). Houses Based on Wood as an Ecological and Sustainable Housing Alternative—Case Study. Sustainability.

[3] Zhen M., Zhang B. (2018). Energy Performance of a Light Wood-Timber Structured House in the Severely Cold Region of China. Sustainability.

[4] (2005). Decree 625 MVaRRSR. Implementing Act No. 555/2005 Coll. on the Energy Performance of Buildings and on the Amendment of Certain Laws (in Slovak Original: Vyhláška 625 MVaRRSR. Ktorou sa Vykonáva Zákon č. 555/2005 Z.z. o Energetickej Hospodárnosti Budov a o zmene a Doplnení Niektorých Zákonov).

[5] Hens L., Hugo S. (2012). Timber-Framed Construction. Performance Based Building Design 2: From Timber-Framed Construction to Partition Walls.

[6] Steeman M., Himpe E., Vanroelen M., Roeck M. (2019). Environmental impact of timber frame walls. IOP Conf. Ser. Earth Environ. Sci..

[7] Woodard A.C., Milner A.C., Khatib J.M. (2016). Sustainability of timber and wood in construction. Sustainability of Construction Materials.

[8] Nowak H., Nowak Ł. (2021). Non-Destructive Possibilities of Thermal Performance Evaluation of the External Walls. Materials.

[9] Desogus G., Mura S., Ricciu R. (2011). Comparing different approaches to in situ measurement of building components thermal resistance. Energy Build..

[10] Soares N., Martins C., Gonçalves M., Santos P., da Silva L.S., Costa J.J. (2019). Laboratory and in-situ non-destructive methods to evaluate the thermal transmittance and behavior of walls, windows, and construction elements with innovative materials: A review. Energy Build..

[11] Iringová A., Vandličková D., Diviš M. (2019). Impact of the Fire and Acoustic Protection on the Composition of Lightweight Wood-based Cladding Envelopes in the Construction of Apartment Buildings in Passive Standard. IOP Conf. Ser. Mater. Sci. Eng..

[12] Niziurska M., Wieczorek M., Borkowicz K. (2022). Fire Safety of External Thermal Insulation Systems (ETICS) in the Aspect of Sustainable Use of Natural Resources. Sustainability.

[13] Michalak J., Czernik S., Marcinek M., Michałowski B. (2020). Environmental burdens of External Thermal Insulation Systems. Expanded Polystyrene vs. Mineral Wool: Case Study from Poland. Sustainability.

[14] Iringová A. (2018). Design of envelopes for timber buildings in terms of sustainable development in the low-energy construction. IOP Conf. Ser. Mater. Sci. Eng..

[15] Yin C., Zheng Q., Zeng J., Yang J., Xiao J. (2015). Composite sandwich panel with multifunction of load bearing, heat insulation, and thermal protection. J. Compos. Mater..

[16] Slimani Z., Trabelsi A., Virgone J., Zanetti Freire R. (2019). Study of the Hygrothermal Behavior of Wood Fiber Insulation Subjected to Non-Isothermal Loading. Appl. Sci..

[17] Asli M., Sassine E., Brachelet F., Antczak E. (2021). Hygrothermal behavior of wood fiber insulation, numerical and experimental approach. Heat Mass Transf..

[18] Korjenic A., Klarić S., Hadžić A., Korjenic S. (2015). Sheep Wool as a Construction Material for Energy Efficiency Improvement. Energies.

[19] Zach J., Korjenic A., Petránek V., Hroudova J., Bednar T. (2012). Performance evaluation and research of alternative thermal insulations based on sheep wool. Energy Build..

[20] Ďurica P., Iringová A., Ponechal R., Rybárik J., Vertal M. (2017). Energy and Environmental Design and Evaluation of Buildings (in Slovak Original: Energetické a Environmentálne Navrhovanie a Hodnotenie Budov).

[21] Ďurica P., Grúňová Z., Ponechal R., Rybárik J., Vertal M. (2015). Building Pathology (in Slovak Original: Patológia Budov).

[22] Lokaj A., Gocál J., Ďurica P. (2010). Wooden Buildings and Structures I. and II. (in Slovak Original: Dřevostavby a Dřevěné Konstrukce I a II).

[23] Bomberg M. (1980). Blown Mineral Fiber Insulation.

[24] Evans M. (2014). Unbonded Loosefill Insulation System. U.S. Patent.

